# Using Data Mining Approach for Student Satisfaction With Teaching Quality in High Vocation Education

**DOI:** 10.3389/fpsyg.2021.746558

**Published:** 2022-01-20

**Authors:** Bailin Chen, Yi Liu, Jinqiu Zheng

**Affiliations:** ^1^Department of Student Affairs, Dongguan Polytechnic, Dongguan, China; ^2^Department of Computer Engineering, Dongguan Polytechnic, Dongguan, China; ^3^Department of Biomedical Engineering, Guangdong Medical University, Dongguan, China

**Keywords:** artificial intelligence, data mining approach, high vocation education, teaching quality, student satisfied

## Abstract

High vocation education is an important foundation for China to cultivate high teaching quality and technical and skilled talents. In the new era, the acceleration of the development of modern vocational education is put in a more prominent position. It is proposed that we should adhere to moral education, closely combine this with the needs of technological change and industrial upgrading, constantly improve the quality of high vocational education teaching, and cultivate more technical and skilled talents with both political integrity and ability for modernization construction. Under the background of social informatization, using artificial intelligent technology to solve these problems can play an important role for improving the teaching quality of high vocational education. This paper proposed a data mining approach in promoting student satisfaction with the teaching quality of high vocational education. We design a questionnaire for Students’ satisfaction with the teaching quality of basic entrepreneurship curriculum. We take the survey data of vocation education as an example and use mining technology analysis software to understand the current status of the teaching quality of basic entrepreneurship curriculum. The results determine the main factors affecting Students’ satisfaction with teaching quality. The results of this paper can be used in student management, education strategy, student education satisfaction, and teaching quality in high vocation college education, and to improve the teaching quality of fundamentals of entrepreneurship curriculum in high vocation education.

## Introduction

Vocational education is an important part of the national education system and human resource development. The motion of higher vocational colleges should seize opportunities, adhere to moral education, deepen the reform and innovation of teaching methods, improve teaching quality, and cultivate more high-quality, technically skilled talents. In the era of social information, the application of data mining technology to the evaluation of teaching quality in higher vocational colleges has gradually become an important way for higher vocational colleges to improve the quality of teaching and talent training (Liu, 2019; Dai, 2020).

With the rapid development of computer and network technology, new media tools such as Weibo, WeChat, QQ, Douyin, etc. emerge endlessly and iteratively. To a certain extent, the Internet has replaced traditional media such as newspapers and television and has become indispensable in people’s daily lives as a source of information. However, while the Internet has brought infinite convenience to people’s access to information, it has also brought some problems, the most important of which is the lack of supervision and filtering, coupled with the high degree of disorder of information resources on the Internet, and the existence of large amounts of duplication and uselessness. Information has brought difficulties to people using the Internet, that is, people cannot effectively select and use the information resources they need in the complex network world. Humans urgently need to find a way to quickly and accurately discover what they need from the massive information resources on the Internet, and a way to extract effective information. In order to solve this problem, data mining technology is used (Song, 2018; Ye, 2020).

The first section of this paper mainly introduces the relevant research background, data mining technology, and the idea of conducting research on the basic course of entrepreneurship for vocational students; section “Relative Work” analyzes the survey data using data mining methods; section “Research Method and Data Analysis” summarizes the influence on the teaching of basic entrepreneurship for vocational students; the main factor of satisfaction. Section “Results and Analysis” elaborates on the conclusion and suggestions to improve the basic course of entrepreneurship for vocational students.

## Relative Work

### Data Mining

**FIGURE 1:**
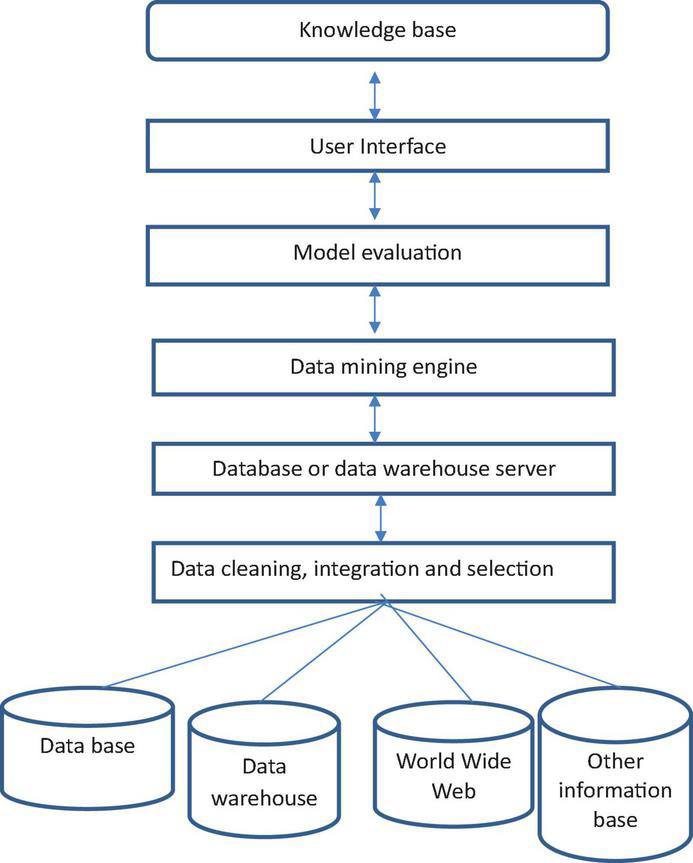
Roadmap of data mining technology.

Data mining (DM), also known as knowledge discovery in database (KDD), refers to the comprehensive use of association analysis, cluster analysis, deviation analysis, classification, prediction, and time series based on database technology. Models and other methods are the process of revealing hidden, unknown, and potentially valuable information and knowledge from a large amount of incomplete, noisy, fuzzy, and random actual data. Data mining is a decision support process, which is mainly based on artificial intelligence, machine learning, pattern recognition, statistics, databases, visualization technology, etc., which can analyze data with a high degree of automation, make inductive reasoning, extract effective data from it (Wang, 2020; Wang and Guo, 2020), and shorten the time for people to browse repeated information, so that people can find and effectively use network information resources in a timely manner, help people adjust strategies, reduce risks, and make correct decisions (Li, 2017; Xin, 2021). The roadmap of data mining technology is shown in Figure 1.

The entrepreneurship basic course is an entrepreneurial enlightenment education course offered by higher vocational colleges. The purpose of the course is not to train many successful graduates, but to cultivate Students’ innovative thinking and workplace quality, and to encourage students to actively learn professional knowledge and skills to achieve the goal of enhancing Students’ competitiveness in the workplace and the quality of employment. Student satisfaction with teaching is an important indicator to measure the teaching quality of entrepreneurship basic courses (Wei and Zhang, 2019; Ke and Chen, 2021). The author took this as a starting point to carry out a questionnaire survey of satisfaction with the teaching quality of entrepreneurship basic courses in higher vocational colleges, then analyzed this information by using data mining methods, and summarized the basic courses of entrepreneurship. The status of teaching quality, to find out the main factors affecting teaching quality, was used as a reference basis for improving teaching suggestions and countermeasures (Hou and Vijayalakshmi, 2020; Zhu, 2021).

### Student Satisfaction Method

The different applications used in universities could directly interfere with both academic performance and student satisfaction. The effectiveness and satisfaction was more in control with technology, and they used a combined dataset in effective measure by self-evaluation assessment (Tawafak et al., 2018a; Ma, 2020). There are multiple innovative performances that have been developed to connect students with MOOC in science education, and the development gives students the necessary skills and knowledge to assist faculty members (Humphreys and Gaston, 2016; Tawafak et al., 2018b). Between the faculty and learners, this difference of emerging e-learning systems can be adapted for managing online courses like the MOOC system (Zhang and Wu, 2018; Long, 2019). These types of system can help methodology improvement through different types of analysis of student actions, and students at risk to assess achievements of learning goals (Guo, 2021).

There are two main options for assessment and satisfaction; one is called automated machine grading, which is suitable for grading quizzes and calculations if the outcome is well defined by use of course content and teacher knowledge. The second option is peer grading using an interactivity factor where MOOC participants evaluate several achievements of other students and provide feedback on their quality and correctness. There are many key elements of these technologies, such as providing all content, assessment on the same platform, auto-graded assessment for immediate feedback, and discussion forums for questions and answers. Finally, these studies still work individually based on their factors but not in combination with a technology acceptance model and learning processing requirements.

The computations were based on the interactivity and effectiveness of comprehensive factors for students and academic performance that has been adopted as new technologies. This study is to establish functions that have affected teaching assessment and academic performance. In addition, the network incorporates three keys of self-learning namely interactivity, technology integration, and personal factors. The method uses a questionnaire built on three factors of self-management, desire to learn, and self-control by use of a newly proposed multiple-indicator-multiple-cause model. A survey with different users on the MOOC platform was used to determine the intention of using the model. The result was significantly influenced by perceived usefulness, perceived ease of use, and user satisfaction based on academic performance (Cui and Ma, 2020; Nowakowska et al., 2020).

## Research Method and Data Analysis

### Research Method

The author uses SPSS statistical software for data mining and analysis, and summarizes the teaching status and quality of entrepreneurship basic courses in higher vocational colleges. The author uses the Likert five-point scale method to investigate the overall satisfaction of students in the basic courses of entrepreneurship in higher vocational colleges, and divides the overall satisfaction evaluation of students into course objectives, number of courses, course content, teaching resources, teacher attitudes, and teaching methods; 10 evaluation indicators, such as teaching effect, are selected from 5 levels of inspection: “very non-conforming” (1 point), “relatively inconsistent,” (2 points), “general” (3 points), “relatively consistent” (4 points), and “very consistent” (5 points). Gender is used as a grouping variable to conduct an independent sample *T*-test on the data of student satisfaction in the basic course of entrepreneurship in higher vocational colleges.

### Data Analysis

The author conducted a student satisfaction survey on entrepreneurship basic courses in vocational colleges, the content structure of the questionnaire is shown in Table 1. A total of 3,231 questionnaires were issued and 2,819 were recovered (recovery rate of 87.24%). There are 2,702 valid questionnaires (effective rate 95.85%). The overall Cronbach coefficient of the valid questionnaire is 0.983, and the Cronbach coefficient of each dimension is above 0.8. The reliability of the questionnaire is good. The effective sample composition is shown in Table 2.

**TABLE 1:** The content structure of the questionnaire of student satisfaction survey on the basic course of entrepreneurship in higher vocational college.

Dimension	Operational definitions	Number of questions
Students’ personal characteristics	Gender, place of origin, grade, and major	4
The value and teaching quality of basic entrepreneurship courses	Cause dimensions: course objectives, course contents, course structure, class schedules, teaching organization, teaching resources, course assessment methods, and course management	14
	Teacher dimensions: knowledge level, teaching methods, interaction between the teacher and students, personal charm, teaching attitude, and classroom management skills	7
	Student dimensions: learning interest, learning style, learning engagement, course participation, learning atmosphere, and group interaction	8
Overall satisfaction with basic entrepreneurship courses	Course number, class schedules, course objectives, course contents, course structure, course evaluation, teaching resources, teaching methods, teaching management, and teaching effect	Scale (12)
Overall satisfaction of professional courses	Course number, class schedules, course objectives, course contents, course structure, course evaluation, teaching resources, teaching methods, teaching management, and teaching effect	Scale (15)
Open questions	Suggestions, contact information	2
Number of questions		62

**TABLE 2:** The composition of valid samples of the questionnaire on student satisfaction in the basic course of entrepreneurship in higher vocational colleges.

Variables	Category	Number of people	Percentage (%)
Gender	Male	1,896	70.1
	Female	806	29.9
Grade	Grade 2018	875	32.4
	Grade 2019	1,052	38.9
	Grade 2020	775	28.7
Subject concerned	Science	333	12.4
	Engineering	1,687	62.4
	Humanities and social sciences	579	21.4
	Others	103	3.8
Origin of place	Cities	1,056	39.1
	Rural areas	1,650	60.9

### Analysis of Data Mining Methods

#### The Overall Satisfaction of Students in the Basic Course of Entrepreneurship Is Positively Low

The author uses the Likert five-point scale method to investigate the overall satisfaction of students in the basic courses of entrepreneurship in higher vocational colleges, and divides the overall satisfaction evaluation of students into course objectives, number of courses, course content, teaching resources, teacher attitudes, and teaching methods; 10 evaluation indicators, such as teaching effect, are selected from 5 levels of inspection: “very non-conforming” (1 point), “relatively inconsistent,” (2 points), “general” (3 points), “relatively consistent” (4 points), and “very consistent” (5 points). In addition, the questionnaire also carried out an additional survey on the overall satisfaction of students in professional courses (Huang et al., 2019; Chen et al., 2020).

**FIGURE 2:**
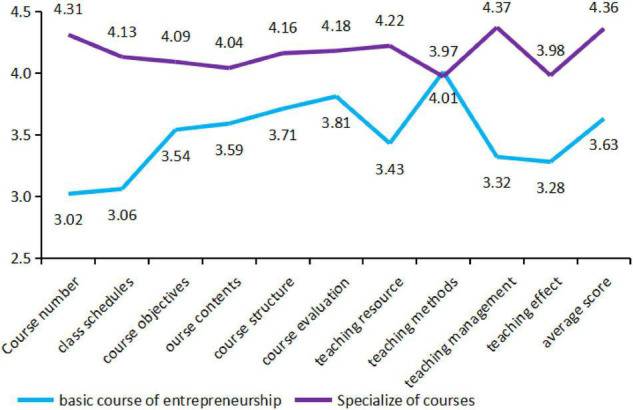
Comparative analysis of student satisfaction between entrepreneurship basic courses and specialized courses.

It can be seen from Figure 2 that the surveyed Students’ satisfaction with basic entrepreneurship courses and professional courses are both positively satisfied. The satisfaction of Xiang is low (average score: 3.29 points, full score of 5), and the satisfaction of professional courses is significantly higher than that of entrepreneurial basic courses. But in terms of “teacher attitude,” the basic course of entrepreneurship (4.01 points) is slightly higher than the professional course (3.97 points). Among the 10 indicators specifically examined in the basic course of entrepreneurship, the highest student satisfaction is “teacher attitude,” the lowest indicator is “number of courses offered,” followed by “class schedule” and “teaching methods,” and overall satisfaction with the remaining indicators is similar (Huang et al., 2017; Chen et al., 2019).

#### Gender and Grade Have No Significant Influence on the Satisfaction of Students in Basic Entrepreneurship Courses

Using gender as a grouping variable to conduct an independent sample *T*-test on the data of student satisfaction in the basic course of entrepreneurship in higher vocational colleges, it can be found that from the mean result, the overall satisfaction of girls (3.43 points) is slightly higher than that of boys (3.15 points). However, under the condition of homogeneity of variance, the significance of the mean test is greater than 0.05. Therefore, the gender variable has no significant impact on the overall satisfaction of students in the basic course of entrepreneurship (Guo, 2020). At the same time, the satisfaction *P*-values of students of different genders in the structural variables of basic entrepreneurship courses are also greater than 0.05, indicating that in the current satisfaction evaluation of basic entrepreneurship courses in higher vocational colleges, gender is not the main influencing factor, and students of different genders are satisfied with basic entrepreneurship courses (Chen et al., 2015; Huang et al., 2015). The independent sample test results of degree are shown in Table 3.

**TABLE 3:** Independent sample test of satisfaction of students of different genders with basic entrepreneurial courses.

		Levene test of variance equation		*T*-test of mean value equation					
		*F*	Sig.	*T*	Sig.(2-lateral)	Mean difference	Standard error	95% confidence interval of the difference	
								Lower limit	Upper limit
Course number	Assuming equal variable Assuming unequal variable	4.298	0.039	−1.612 −1.612	0.099 0.099	−0.224 −0.224	0.131 0.131	−0.502 −0.502	0.031 0.031
Class schedules	Assuming equal variable Assuming unequal variable	1.496	0.217	−0.283 −0.283	0.769 0.769	−0.044 −0.044	0.121 0.121	−0.247 −0.247	0.189 0.189
Cause objectives	Assuming equal variable Assuming unequal variable	0.740	0.392	−0.646 -0.646	0.528 0.528	−0.075 −0.075	0.122 0.122	−0.288 −0.288	0.152 0.152
Cause contents	Assuming equal variable Assuming unequal variable	0.378	0.539	−0.551 −0.551	0.582 0.582	−0.060 −0.060	0.109 0.109	−0.274 −0.274	0.154 0.154
Cause structure	Assuming equal variable Assuming unequal variable	5.345	0.034	−1.315 −1.315	0.303 0.303	−0.151 −0.151	0.121 0.121	−0.372 −0.372	0.092 0.092
Course evaluation	Assuming equal variable Assuming unequal variable	0.002	0.989	0.368 0.368	0.688 0.688	0.040 0.040	0.120 0.120	−0.179 −0.179	0.251 0.251
Teaching resources	Assuming equal variable Assuming unequal variable	1.009	0.317	0.241 0.241	0.821 0.821	0.025 0.025	0.114 0.114	−0.192 −0.192	0.252 0.252
Teacher’s attitude	Assuming equal variable Assuming unequal variable	0.849	0.347	0.229 0.229	0.822 0.822	0.027 0.027	0.114 0.114	−0.198 −0.198	0.251 0.251
Teaching methods	Assuming equal variable Assuming unequal variable	0.196	0.647	0.159 0.159	0.859 0.859	0.021 0.021	0.117 0.117	−0.218 −0.218	0.238 0.238
Teaching effect	Assuming equal variable Assuming unequal variable	1.022	0.324	0.241 0.241	0.795 0.795	0.101 0.101	0.122 0.122	−0.211 −0.211	0.248 0.248

A one-way analysis of variance was carried out on the satisfaction of students in the basic course of entrepreneurship with grade as a factor. The results showed that the *P*-value of Students’ satisfaction with each structural index of the course was greater than 0.05, and the variance was homogeneous, and one-way analysis of variance could be carried out. In the follow-up one-way analysis of variance results, the satisfaction *P*-value of only the “teaching method” is 0.044, which is less than the significance level of 0.05, indicating that the grade has a significant impact on the Student’s satisfaction with the teaching methods of entrepreneurship basic courses (Liu et al., 2014; Lin et al., 2020). However, in the subsequent multiple comparative analysis, the significance of the comparison of satisfaction with the basic course of entrepreneurship among students of different grades is greater than 0.05, and the difference is not significant. Therefore, the comprehensive judgment of the grade has no significant effect on the satisfaction of students in the basic course of entrepreneurship. The multiple analysis of Students’ satisfaction with teachers’ teaching in different grades is shown in Table 4.

**TABLE 4:** Multiple analysis of Students’ satisfaction with teachers in different grades.

Dependent variable			Mean difference (I-J)	Standard error	Significance	95% confidence interval	
						Lower limit	Upper limit
Satisfaction with teaching method	Grade 2018	Grade 2017	0.321	0.145	0.177	−0.03	0.62
		Grade 2016	0.321	0.146	0.184	−0.08	0.73
	Grade 2017	Grade 2018	–0.317	0.139	0.165	−0.67	0.07
		Grade 2016	0.002	0.155	1.010	−0.43	0.42
	Grade 2016	Grade 2018	–0.345	0.177	0.456	−0.70	0.07
		Grade 2017	–0.001	0.136	1.002	−0.29	0.45
							

#### Different Majors Have a Significant Impact on the Satisfaction of Students in Basic Entrepreneurship Courses, and Engineering Students Have the Highest Satisfaction

A one-way analysis of variance was carried out on the student satisfaction of the basic course of entrepreneurship with the major as a factor. The results showed that the *P*-values of student satisfaction in each structural index of the course were all greater than 0.05. The variance was homogeneous, and the one-way analysis of variance could be carried out. In the follow-up one-way analysis of variance results, the *P*-values of Students’ satisfaction with teaching methods and teaching attitudes were 0.006 and 0.041, respectively, which were less than the significant level of 0.05, indicating that majors had a significant impact on student satisfaction with teaching methods and teaching attitudes. According to the subsequent multiple comparative analysis, there is a significant difference in the satisfaction of engineering majors and science majors in teachers’ classroom teaching and teaching management. Combined with the mean statistical analysis, it can be found that engineering students are highly satisfied with teachers’ teaching methods and teaching attitudes. The multiple analysis of Students’ satisfaction with the teaching of basic entrepreneurship courses in different majors is shown in Table 5.

**TABLE 5:** Multiple analysis of satisfaction with the teaching of entrepreneurship basic courses by students of different majors.

Dependent variable			Mean difference (I-J)	Standard error	Significance	95% confidence interval of the difference	
						Lower limit	Upper limit
Students’ satisfaction with teaching methods	Engineering	Science	0.592*	0.178	0.004	0.17	1.15
		Humanities and social sciences	0.103	0.141	1.020	–0.24	0.37
		Others	0.273	0.534	1.002	–1.25	1.67
	Science	Engineering	−0.671*	0.235	0.002	–1.13	–0.14
		Humanities and social sciences	–0.540	0.210	0.052	–1.21	0.03
		Others	–0.391	0.553	1.001	–1.81	1.12
	Humanities and social sciences	Engineering	–0.121	0.124	1.003	–0.42	0.24
		Science	0.547	0.210	0.053	–0.02	1.12
		Others	0.151	0.577	1.010	–1.22	1.63
	Others	Engineering	–0.268	0.546	1.064	–1.13	1.42
		Science	0.387	0.572	1.040	–1.70	1.78
		Humanities and social sciences	–0.145	0.432	1.020	–1.21	1.26
Students’ satisfaction with teacher’s attitude	Engineering	Science	0.542*	0.185	0.047	0.05	1.02
		Humanities and social sciences	0.188	0.171	1.001	–0.11	0.66
		Others	0.154	0.511	1.007	–1.44	1.68
	Science	Engineering	−0.536*	0.295	0.037	–1.05	–0.02
		Humanities and social sciences	–0.354	0.221	0.672	–0.94	0.73
		Others	–0.481	0.578	1.070	–1.74	1.78
	Humanities and social sciences	Engineering	–0.183	0.141	1.000	–0.56	0.19
		Science	0.354	0.221	0.662	–0.23	0.94
		Others	–0.027	0.572	1.000	–1.55	1.49
	Others	Engineering	–0.175	0.582	1.010	–1.75	1.54
		Science	0.371	0.587	1.040	–1.17	1.97
		Humanities and social sciences	0.021	0.577	1.227	–1.41	1.44

*Indicates that the significance level of the mean difference is 0.05.

#### Internal Teaching Factors That Affect the Satisfaction of Students in the Basic Course of Entrepreneurship

With reference to the previous literature research results, the author selected 20 variables in the three dimensions of curriculum, teachers, and students in the section of “Basic Entrepreneurship Course Offering Value and Teaching Quality” as hypothetical variables for the internal influencing factors of student satisfaction in basic entrepreneurship courses. A multiple regression analysis centered on “satisfaction with specific entrepreneurial basic courses” found that the first to enter the regression model were six variables: teaching method, teacher attitude, course difficulty, course assessment method, learning interest, and learning input. The *T*-test probability values of the remaining variables are all greater than 0.05, so the “linear regression model” cannot be introduced, so they are eliminated. In the model summary table shown in Table 6, model 6 including these six factors has the largest adjustment R^2^ and the best fit, so this model is selected as the main object of subsequent analysis.

**TABLE 6:** Summary of multiple regression analysis models for internal influencing factors of student satisfaction in entrepreneurship basic course.

Model	*R*	*R* ^2^	Adjusted *R*^2^	Standard error of estimation	Revised statistics				
					*R*^2^ revision	*F* revision	df1	df2	Sig. *F* revision
1	0.829a	0.638	0.627	4.176	0.678	610.717	1	298	0.001
2	0.843b	0.721	0.757	3.552	0.022	39.634	1	236	0.001
3	0.869c	0.771	0.779	3.343	0.034	19.913	1	224	0.001
4	0.847d	0.814	0.797	3.245	0.014	5.674	1	252	0.015
5	0.845e	0.827	0.808	3.224	0.007	4.404	1	271	0.039
6	0.901f	0.780	0.812	3.204	0.006	4.613	1	270	0.012

According to the results of the ANOVA analysis of variance shown in Table 7, the “regression sum of squares” of the model consisting of six variables: teaching method, teacher attitude, course difficulty, course assessment method, learning interest, and learning input is 12686.160, and the “total sum of squares” is 15660.987, so this linear regression model explains 81% of the total sum of squares. At the same time, with the introduction of various factors, the significance probability value of the “F” statistic behind each model in the ANOVA analysis results is always 0, less than 0.01, so the null hypothesis that the overall regression coefficient is 0 can be rejected. So far, it can be concluded that there is a linear relationship between student satisfaction of a single basic course of entrepreneurship and teaching methods, teacher attitudes, course difficulty, course assessment methods, learning interest, and learning input.

**TABLE 7:** Multiple regression analysis of internal influencing factors of student satisfaction in the basic course of entrepreneurship ANOVA analysis of variance.

Model		Sum of squares	df	Mean square	*F*	Sig.
6	Return	13495.140	8	1425.573	157.712	0.104
	Residual error	3201.831	294	10.368		
	Total	15774.977	279			

In the coefficient table of the selected model (Table 8), the tolerance of the collinearity statistics of each factor is greater than 0.1, and the VIF is less than 5, and there is no characteristic value that can explain all of them at the same time in the subsequent collinearity diagnosis. So there is no collinearity among the above six variables. In addition, since the Sig value of the constant item in the coefficient table is 0.115, which is greater than 0.1, which is not significant, the standardized regression equation can be derived from the standard coefficients in the table: Satisfaction of a single entrepreneurial foundation course = 0.225* teaching method + 0.211* teaching Attitude + 0.136*course difficulty + 0.124*assessment method + 0.111*learning interest + 0.058*learning input. It can be seen that there is a linear relationship between the six variables of student satisfaction and teaching methods, teacher attitude, course difficulty, course assessment method, learning interest, and learning input in a single basic course of entrepreneurship, and the degree of influence decreases successively.

**TABLE 8:** Multivariate regression analysis model coefficients of internal influencing factors of student satisfaction in the basic course of entrepreneurship.

Model		Non-standardized coefficient		Standard coefficient	T	Sig	Collinearity statistics	
		B	Standard error	Trial version			Tolerance	VIF
6	(Constant)	1.638	1.038		1.585	0.214		
	Teaching method	1.597	0.419	0.223	4.208	0.010	0.289	4.367
	Teacher’s attitude	0.871	0.296	0.213	3.005	0.001	0.231	2.045
	Course difficulty	0.979	0.284	0.126	3.432	0.000	0.406	1.934
	Examination method	0.988	0.273	0.114	3.572	0.000	0.452	2.237
	Learning interest	0.778	0.360	0.121	2.464	0.024	0.324	3.125
	Learning engagement	0.357	0.175	0.068	2.145	0.012	0.967	1.223

## Results and Analysis

### The Student Satisfaction of the Basic Courses of Entrepreneurship in Higher Vocational Colleges Is Generally Positively Low

According to the data mining and analysis of the questionnaire, students in higher vocational colleges are at a low level of positive satisfaction with the structural variables and overall satisfaction of the entrepreneurial foundation course. Among them, the overall evaluation of the number of courses is the lowest, followed by the class schedule and teaching methods. The overall evaluation of entrepreneurship is relatively low, and the three together constitute the bottom of the evaluation of basic entrepreneurship courses. Compared with professional courses, the overall satisfaction of basic entrepreneurship courses is lower.

### The Origin of Students and Majors Have a Significant Impact on the Satisfaction of Students in the Basic Courses of Entrepreneurship

From the perspective of Students’ personal characteristics, the factors that have a significant impact on the satisfaction of basic entrepreneurship courses are the origin of students and their major. In terms of student origin, students from cities and towns are more satisfied with the content of basic entrepreneurship courses than students from rural areas, which may be related to the higher learning expectations of rural students for basic entrepreneurship courses. As far as majors are concerned, engineering Students’ satisfaction with course teaching methods and teaching attitudes is significantly higher than that of science majors and liberal arts majors. This may be because the research sample selected by the author has a certain degree of engineering bias in the subject setting, which has a certain indirectly impact on student satisfaction.

### The Teaching Methods and Teaching Attitudes of Teachers Are the Most Significant Factors That Affect the Satisfaction of Students in the Basic Course of Entrepreneurship

The teaching methods adopted by the teachers and their own teachers’ attitudes are the two variables that have the greatest impact on student satisfaction in the basic course of entrepreneurship. As an important factor in curriculum practice, the teaching methods designed and used by teachers not only affect Students’ learning enthusiasm and learning input, but also affect the transmission of knowledge and values carried by the curriculum content. Teachers’ teaching attitudes also have an important impact on student satisfaction. Teachers’ good teaching attitudes can be perceived by students during the teaching process, and then form their own teaching style and teaching charm, which affects Students’ satisfaction with the course.

### Students’ Learning Interest and Learning Investment Have a Significant Impact on the Satisfaction of Basic Entrepreneurship Courses

As far as learning interest is concerned, regardless of whether the Students’ learning interest stems from their own interest in the content of the basic entrepreneurship course, or they are inspired by the teacher’s teaching during the course of learning the basic entrepreneurship course, their interest in learning has a considerable impact on the satisfaction of students in the basic entrepreneurship course. The latter is also closely related to the teacher factor in a previous article. At the same time, learning investment also has a significant impact on Students’ satisfaction with the basic courses of entrepreneurship. Learning investment is not only related to Students’ learning interests, but also closely related to factors such as curriculum content, curriculum assessment, curriculum value, teaching mode, teaching attitude, and teacher charm.

## Conclusion and Suggestions

### Conclusion

This paper’s contributions and suggestions include:

(1). Increase the number of basic entrepreneurship courses and establish a sound course classification system. Restricted by factors such as the orientation of school development goals, the distribution of disciplines, the level of teachers, and the total number of class hours, the number of courses in basic entrepreneurship courses in higher vocational colleges is generally relatively small, which directly leads to relatively insufficient teaching resources for innovation and entrepreneurship education. In addition, most of the current basic courses of entrepreneurship in higher vocational colleges are usually classified as public courses, and the school-wide unified “course-selection-course” approach is adopted, which results in a lack of necessary professional adaptability. One of the ways to solve this problem is to establish and improve the curriculum classification system. Schools can combine the learning needs of different majors and different grades, centering on “problem-oriented learning,” and scientifically classify the basic courses of entrepreneurship in the form of curriculum modules. And use grading to meet the development needs of students of different majors.
(2). Strengthen the construction of the faculty of basic entrepreneurship courses, and establish and improve related systems. The teaching quality of the basic course of entrepreneurship is directly related to the teaching quality of the course. Teachers in the course should consciously improve their innovation and entrepreneurship through reflection, research, communication, and learning. First of all, in view of the current situation of the lack of teachers for innovation and entrepreneurship courses in higher vocational colleges, the school should select teachers with academic influence, personal charm, and high-level teaching ability to take the lead in forming a teaching team and focus on creating quality courses. Secondly, in view of the current situation of an insufficient reserve of teachers for innovation and entrepreneurship courses in higher vocational colleges, it is necessary to increase efforts to cultivate young teachers and establish a teacher training mechanism to fully tap into the potential advantages of reserve forces. Thirdly, in view of the current situation of imperfect external communication mechanism for teachers of innovation and entrepreneurship courses in higher vocational colleges, it is necessary to explore the establishment of an inter-school mutual assistance exchange platform to realize the “resource sharing” and “external introduction and internal support” of high-quality curriculum teachers. Famous experts and scholars from various schools and research institutions can serve as the main lecturers of the courses, participate in the construction of the course teaching system, and enhance the teaching effect.
(3). Optimize course teaching methods and enrich teaching models. The teacher’s teaching method is the most important factor that affects the satisfaction of students in the basic course of entrepreneurship. Investigation and research found that most of the basic courses of entrepreneurship in higher vocational colleges adopt a teaching model that combines PPT-based teacher teaching and classroom questioning, while most of the teaching models that make students respond positively and impressively include teachers’ ability to adopt humorous language pairs. The knowledge is explained in a simple way, and teachers can apply and practice the curriculum knowledge. It can be seen that the effective interaction between teaching and learning can effectively improve the satisfaction of students in the basic courses of entrepreneurship. Therefore, teachers need to consciously strengthen the situation design, create conditions, set tasks, and collectively operate with team members during curriculum design, such as manual entrepreneurship sandbox practice, electronic entrepreneurship sandbox practice, etc., and multimedia case analysis can be used in specific links. Participatory group discussions and other methods enable active interaction between teachers and students, and between students and students, and create innovative and entrepreneurial thinking.

## Data Availability Statement

The original contributions presented in the study are included in the article/supplementary material, further inquiries can be directed to the corresponding author/s.

## Author Contributions

BC: conceptualization, methodology, validation, investigation, and writing. YL: funding acquisition, formal analysis, software, resources, and visualization. JZ: funding acquisition, methodology, validation, writing -review and editing, and supervision. All authors contributed to the article and approved the submitted version.

## Conflict of Interest

The authors declare that the research was conducted in the absence of any commercial or financial relationships that could be construed as a potential conflict of interest.

## Publisher’s Note

All claims expressed in this article are solely those of the authors and do not necessarily represent those of their affiliated organizations, or those of the publisher, the editors and the reviewers. Any product that may be evaluated in this article, or claim that may be made by its manufacturer, is not guaranteed or endorsed by the publisher.
